# Setting of Methods for Analysis of Mucosal Antibodies in Seminal and Vaginal Fluids of HIV Seropositive Subjects from Cambodian and Italian Cohorts

**DOI:** 10.1371/journal.pone.0009920

**Published:** 2010-03-29

**Authors:** Carla Donadoni, Cinzia Bisighini, Lorenza Scotti, Lorenzo Diomede, Marie Ngyen, Janin Nouhin, Lucia DeSantis, Antonella Zambon, Davide Ferrari, Giulia Gallotta, Giovanni Corrao, Gianfranco Pancino, Lucia Lopalco

**Affiliations:** 1 Division of Immunology, Infectious Diseases and Trasplantation, San Raffaele Scientific Institute, Milan, Italy; 2 Department of Statistics, University of Milano-Bicocca, Milan, Italy; 3 HIV/Hepatitis Laboratory, Institut Pasteur du Cambodge, Phnom Penh, Cambodia; 4 Department of Obstetrics and Gynaecology, University Vita Salute, San Raffaele Scientific Institute, Milan, Italy; 5 Department of Obstetrics and Gynaecology, San Raffaele Scientific Institute, Milan, Italy; 6 Department of Infectious Diseases, San Raffaele Scientific Institute, Milan, Italy; 7 Regulation of Retroviral Infections Unit, Institut Pasteur, Paris, France; University of Toronto, Canada

## Abstract

**Background:**

Genital mucosae play a key role in protection from STD and HIV infection, due to their involvement in both horizontal and vertical disease transmission. High variability of published observations concerning IgA isolation and quantification underlies the strong requirement of specific methods able to maximize investigation on HIV-specific IgA.

**Methodology:**

Genital fluids from 109 subjects, including male and female cohorts from Italy and Cambodia, were collected, aliquoted and processed with different techniques, to assess optimal conditions maximizing mucosal antibody recovery. Three sampling techniques, up to sixteen preservation conditions, six ELISA methods and four purifications protocols were compared.

**Principal Findings:**

The optimal method here described took advantage of Weck-Cel sampling of female mucosal fluids. Immediate processing of genital fluids, with the addition of antibiotics and EDTA, improved recovery of vaginal IgA, while the triple addition of EDTA, antibiotics and protease inhibitors provided the highest amount of seminal IgA. Due to low amount of IgA in mucosal fluids, a high sensitive sandwich ELISA assay was set; sensitivity was enhanced by milk-based overcoating buffer and by a two-step biotin-streptavidin signal amplification. Indeed, commercial antisera to detect human immunoglobulins showed weak cross-reactivity to different antibody types. Three-step affinity purification provided reproducible immunoglobulin recovery from genital specimens, while conventional immuno-affinity IgA purification was found poorly manageable. Affinity columns were suitable to isolate mucosal IgA, which are ten-fold less concentrated than IgG in genital specimens, and provided effective separation of IgA monomers, dimers, and J-chains. Jacalin-bound resin successfully separated IgA1 from IgA2 subfraction.

**Conclusions/Significance:**

Specific, effective and reliable methods to study local immunity are key items in understanding host mucosal response. The sequence of methods here described is effective and reliable in analysing humoral local responses, and may provide a solid advance to identify and measure the effective mucosal responses to HIV.

## Introduction

In the majority of cases, if not in all, HIV infection takes place through the mucosal route, i.e. by sexual contact or child delivery [1]. Genital mucosae are the target districts where early immune response to HIV are likely to take place [2]; consequently, no advance in control or prevention of the early phases of HIV infection can be achieved without acquiring deep knowledge on local innate and adaptive responses [3].

Several investigators have reported the induction of humoral responses and of neutralizing antibodies to HIV, both in systemic and in mucosal compartments, while other laboratories failed in observing similar responses [4]–[6]. No prevalent mechanisms of protective immune response to HIV have been by far identified. Experimental challenges with SIV and immunotherapy of HIV-positive patients did show the effectiveness of systemic and mucosal humoral responses, and especially that of neutralizing antibodies [7]–[10]. Mucosal responses observed in HIV-positive and HIV-exposed subjects often show great heterogeneity; this finding may depend on individual variability or on modes of virus exposure [3], [11], but it may also reflect the intrinsic difficulty to evaluate mucosal immunity and to measure local humoral response.

Antibodies isolated from mucosal compartments may originate from systemic and/or from local cells: for example, intestinal fluids are rich in IgA from local cells, while male and female genital fluids mostly contain IgG of systemic origin [12], [13]. IgA immunoglobulins from genital fluids present lower concentrations than IgG, and therefore IgA can easily go undetected by standard methods, which are optimized for serum immunoglobulins [14]. This should not be surprising, because antibody concentrations in serum are higher than those found in mucosal secretions [15]. A further factor complicating mucosal fluids analysis is the high concentration of interfering proteins and glycans, which can hamper antibodies reactivity and interfere with their isolation. Finally, antibody concentrations in female genital fluids also undergo cyclic variations, according with the phase of menstrual cycle and to hormone levels [16]. Due to the low IgA concentration in genital fluids [17] and to the concurring factors here summarized, it is reasonable that well-working methods suitable for IgG isolation often fail in retrieving mucosal IgA [18]. However, any investigation aimed at characterizing mucosal immunoglobulins strongly needs specific and reliable methods to achieve solid and reproducible results [17], [19]. This study was designed to set and validate optimal methods to isolate and quantify IgA from a panel of HIV-positive and -negative genital fluids.

## Materials and Methods

### Ethics Statement

Written informed consent was obtained from all the participants and for all aspects of the study, including the collection of personal data. The study was approved by the institutional review board from San Raffaele Scientific Institute, Milan, Italy.

### Methods design and setting

The high variability of published observations concerning IgA isolation and quantification underlines the strong requirement of a specific method to recover, quantify and process IgA, also applicable to the investigation of HIV-specific IgA [20], [21]. The aim of this work was therefore the design of an optimal method to maximize quantitative IgA recovery from mucosal fluids. At this purpose, genital fluids obtained from healthy people and from a cohort of HIV-positive individuals were used to set and compare analytical protocols and to validate their specificity and reliability.

### Study population

Two different cohorts were studied: the former enrolled Italian female and male subjects and the latter included Cambodian women only. In detail, the first cohort included 23 HIV-seropositive and 23 healthy control women, enrolled at the San Raffaele Scientific Institute of Milan. The Italian cohort also enrolled 33 healthy and 10 HIV-seropositive Italian men. Genital fluids (vaginal and seminal) were obtained from healthy individuals undergoing routinary control visits (n = 23 females), subjects undergoing assisted fertilization practices (n = 33 males) and from a cohort of HIV+ people enrolled at San Raffaele Infectious Diseases Clinic (n = 33, 23 females and 10 males). Specimens from healthy subjects were used in the preliminary phases of the study, to optimize and compare the effectiveness of experimental conditions, sampling and processing methods.

The second cohort, including 10 HIV-seropositive and 10 healthy Cambodian women, was enrolled at the Pasteur Institute of Cambodia.

All Italian seropositive patients had received antiretroviral therapy for at least 1 year at the time of the study; CD4 cells counts were in the range 322−745×10^3^ cells/mL (median 552) and HIV plasma viraemia was <50 copies/mL in all patients. Female and male healthy controls were matched for age (25–45 years old) and without any known risk factor for HIV infection. Detailed information about sexual behaviour of participants were collected. All participants were asked to avoid sexual intercourses 24–48 hours before the sampling visit; the date of the latest intercourse was reported and samples were controlled by optical microscopy for the presence of spermatozoa. In order to minimize individual variations associated with hormone levels and to enhance antibody recovery [22], all female mucosal samples were obtained during post-ovulatory period (i.e. collected in days 15–20 after the latest menstrual cycle, to ensure that the ovulation has already occurred), in order to minimize individual variability of cervicovaginal secretions that is associated with hormonal levels and menstrual phase [16], [22]. Before assaying for IgA, all samples collected from the cohorts were incubated 30 min at 57°C. This procedure was required to inactivate complement protein cascade, which could interfere with testing, and to inactivate infectious HIV particles, but it did not affect immunoglobulins reactivity.

### Vaginal fluid sampling

Three differrent collection methods were compared:

#### Brushing

Samples of vaginal fluids from healthy women was obtained through extensive brushing of vaginal walls. After sampling, brushes were rinsed in 1 mL PBS and resulting fluids were centrifuged (1,800 g×10 min) to remove epithelial cell debris and immediately put in ice. Within 1 hour from sampling, fluids were sterilized by filtration on 0.22 microm membranes, aliquoted and stored at −80°C.

#### Cervicovaginal Lavage (CVL)

Samples of cervico-vaginal fluids (CVL) from healthy and from HIV+ Cambodian women have been obtained through extensive rinsing of vaginal walls with 7 mL of sterile 1× PBS, dispensed from a sterile syringe directly on vaginal walls. CVL samples were completely recovered with a sterile syringe, immediately refrigerated on ice and centrifuged (1800 g×10 min) to remove epithelial cell debris. Fluids were sterilized by filtration on 0.22 micron membranes, aliquoted and stored at −80°C.

#### Weck-Cel

This method was based on the method described by Coombs et al [21]. Briefly, Weck-Cel sponges (Eyetec Ophthalmic products, Altomed Ltd, UK) were pre-wet in disposable tubes with 50 microL of sterile PBS buffer, and were kept under flow hood, at room temperature until sampling. Sponges were gently inserted in vagina (depth 5–7 cm) and used to rinse throughout the mucosal surface for one minute. No special devices, other than those routinely used in gynecological examinations, were required for Weck-Cel application.

After sampling, sponges heads were placed back in pre-wetting tubes, then were stored at −80∞C until processing. All sponges were carefully weighted before and after the procedure, to determine genital fluid recovery.

### Seminal fluid isolation

Seminal fluids from 33 healthy donors were obtained and processed with the standard procedure of the Centre, to minimize any difference due to processing or maintenance [23]. Samples from ten HIV-positive patients, known to have acquired HIV infection by mucosal route (sexual partners of HIV-positive patients) were collected at San Raffaele Scientific Institute.

Standard protocols require semen incubation (4°C for 1 hr) to reduce sample viscosity, followed by dilution in sterile PBS (1∶2 or 1∶5), gradient centrifugation to recover spermatozoa (1800 g for 10 min), sterile filtration on 0.45 micron filters and complement inactivation (57°C for 30 min), before freezing at −80°C. In order to compare methods and conditions aimed at the optimal recover of mucosal immunoglobulins, each seminal fluid was split in 16 aliquots and assayed as described below.

### Female and Male fluids processing

In order to assess the optimal procedure to preserve immunoglobulins, the efficacy of refrigeration, addition of antibiotics, EDTA and protease inhibition were compared, as unique additive or in double or triple association, as in the list:

- Cryopreservation only
- EDTA 1% V:V
- Protease inhibitor mix 0.1% V:V
- Antibiotic mix 2% V:V
- EDTA 1% V:V + protease inhibitor mix 0.1% V:V
- EDTA 1% + antibiotic mix 2% V:V
- Protease inhibitor mix 0.1% V:V + antibiotic mix 2% V:V
- EDTA 1% + antibiotic mix 4% V:V + protease inhibitor mix 0.1% V:V
- USA guidelines for Mucosal Specimens Sampling and Processing [23]

Final concentrations of reagents examined in the study were: EDTA 0,05 M (to be diluted 1∶100 V:V); a commercial cocktail of Protease Inhibitors (IP) including pepstatin A, E64, bestatin, leupeptin, aprotinin, to be diluted 1∶1000 V:V (SIGMA-ALDRICH Inc, St. Louis, MO, USA); Antibiotics Mix 2%, containing Penicillin 10,000 units, Streptomycin 10 mg/mL and amphoterycin B 25 µg/mL (SIGMA-ALDRICH).

CV fluids were split in seven aliquots and processed/added with a different preservation protocol;

Seminal fluids were split in sixteen aliquots and were processed to reduce viscosity and to preserve antibodies from degradation, according to three major protocols. In the first (“Immediate” mode), the seven reagents were immediately added to fresh semen aliquots before any further procedure. Other seven aliquots received the additional components at the end of the processing, just before the step for complement inactivation (“Delayed” mode). The remaining two aliquots, used as controls, were kept free from additives (“Null”), or were processed according to a published guideline (“Mestecky” protocol) [23].

### ELISA protocols for mucosal IgA/IgG/total Ig quantitation

Six different sandwich ELISA protocols were compared for their efficacy in detecting and quantifying antibodies from mucosal secretions. Methods were set up and compared on a panel of immunoglobulins from genital fluids obtained from healthy people and on standard commercial human immunoglobulins (i.e. single or mixed IgA, IgG and IgM; IgA and IgG from SIGMA-ALDRICH; IgM from CALBIOCHEM, Darmstadt, Germany). All the samples were plated in double replicates. Protocols to be compared were different in the composition of overcoating buffer (BSA 10%+Tween 20 1% vs BSA 10%+Tween 20 5% vs skimmed powdered milk 1%) (SIGMA-ALDRICH) and/or in the revealing agent (biotinylated anti-human immunoglobulins + streptavidin-HRP vs HRP-jacalin).

In detail, ELISA plates (Immuno Plate, F96 Maxysorp, NUNC, Roskilde, Denmark) were coated with a 1∶2,000 dilution of a goat anti-human IgA-IgG-IgM (100 microl/well; KPL, Gaithersburg, MD, USA) and incubated 2 h at room temperature. Purified CVL antibodies and reference standard immunoglobulins (4 nicrog/mL), diluted in the overcoating buffer on a 1∶2 basis, were plated and allowed to react overnight at 4°C.

After extensive washing with PBS 1x-Tween 20 0.1%, plates were incubated 1 h at room temperature with the reagent to be employed (e.g. biotinylated goat anti-human IgA diluted 1∶5,000 V/V in overcoating buffer or jacalin-HRP diluted 1∶500 V/V in overcoating buffer).

ELISA plates incubated with jacalin-HRP (SIGMA-ALDRICH) were directly revealed with commercial TMB Peroxidase Substrate (KPL; 5 minutes of incubation before the addition of H_2_SO_4_ and the spectrophotometric quantification at 450 nm). Sandwich plates incubated with biotinylated antibodies (Goat anti Human IgA/IgG/IgM, SB, Birmingham, AL, USA) required a further incubation with streptavidin-HRP, diluted 1∶3,000 V/V in PBS 1x-Tween 20 0.1% buffer (1 h at room temperature; VECTOR Laboratories, Burlingam, CA, USA) before proceeding with the chromogen reaction.

Quantitative ELISA to measure total mucosal immunoglobulins and IgG were carried out with the optimal protocol set for IgA determination, employing the convenient sandwich reagents (i.e. goat anti-human IgA-IgG-IgM or goat anti-human IgG).

### Immunoglobulin purification

Affinity purification of total immunoglobulins, IgA and IgG fractions from female and male genital fluids was carried out through a sequential automatic chromatography system (Biologic Duoflow, BIO-RAD Laboratories, Hercules, CA, USA), which isolated total immunoglobulins, then IgG and finally IgA fractions from each specimen. Three methods were compared to purify total immunoglobulins, IgG and IgA fractions; all methods employed one, two or three steps of chromatographic separations, such as affinity purification on a specific antibody-bound Sepharose (total Igs, IgG and IgA), affinity purification on Sepharose-Protein A (IgG fraction), anionic exchange chromatography and gel filtration (IgA fraction).

### Sepharose columns setting

CNB-activated Sepharose 4B (GE-Healthcare, Uppsala, SE) was equilibrated in buffer solutions, conjugated with capture antibodies according with manufacturer's instructions and packed in 2 mL or 5 mL Bio-Scale MT columns (BIO-RAD). In detail, Sepharose columns suitable to capture total immunoglobulins, IgA or IgG fractions were prepared from commercial rabbit antibodies recognizing total human immunoglobulins, heavy IgA or IgG heavy chain (SIGMA-ALDRICH). In both cases, a 1∶100 proportion between resin volume and antibody solution was used; bound was allowed, under constant stirring, for three hours at room temperature or overnight at 4°C

After extensive washing with buffer solution, conjugated resin columns were stored at 4°C under PBS 1× buffer containing NaN_3_ 0,05%.

### Affinity purification of total mucosal immunoglobulins

In detail, 100 microL of genital fluids, diluted in phosphate buffer 1x, were applied on 2 mL columns for IgA/IgG purification or on 5 mL column for total Ig purification. Column washing and immunoglobulin elution were carried out according with standard protocols.

Void volumes from IgA column were charged on IgG column and processed with standard methods. Eluted fractions were concentrated with Amicon ultra-centrifuge filter devices (Millipore, Badford, MA, USA), resuspended in a small volume of PBS 1× and sterilized on 0.22 µm membranes before storage at −80°C. Concentration of recovered immunoglobulins were determined by a comparative ELISA assay including standard immunoglobulins dilutions, specifically set up to detect mucosal IgA, as described below.

### IgG purification by Protein G affinity chromatography

IgG fractions from genital fluids were purified by affinity chromatography on HiTrap Protein G HP columns (GE-Healthcare). Protein G from Group G streptococci is known for its binding properties to IgG Fc region, but it is unable to bind IgA and IgM antibodies [24].

In detail, Protein G columns were extensively washed and equilibrated in PBS (1 mL/min flow) before sample application. Sample was applied at 0.5 mL/min; the unbound fraction containing IgA was recovered to be processed conveniently. IgG fraction was eluted with 8 volumes of 200 mM Glycin buffer (pH 2.0; 0,2 mL/min flow) and neutralized at pH 7.0 with 1 M Tris-HCl buffer pH 11.0.

Columns were regenerated with two volumes of elution buffer (1 mL/min flow) and re-equilibrated with PBS buffer (three volumes) before starting a new purification cycle. Both unbound and eluted fractions were added with 0.05% NaN_3_ and stored at 4°C.

### IgM-IgA purification using anion-exchange column chromatography

Residual fractions from IgG purification underwent IgM-IgA purification step, performed by anion exchange column chromatography. After a dialysis step in binding buffer (buffer A: 40 mM NaCl, 20 mM TrisHCl pH 7.2), intended to change saline concentration of the medium, fractions were applied onto the HiTrap Q HP column (GE-Healthcare), previously washed and equilibrated with buffer A. Unbound fractions were recovered and stored; IgA and IgM fractions were eluted from the column with 10 volumes of buffer B (340 mM NaCl, 20 mM TrisHCl, pH 7.2). Columns were regenerated with five volumes of buffer C (1 M NaCl, 20 mM Tris-HCl, pH 7.2), then re-equilibrated with buffer A (five volumes) before starting a new purification cycle. Both unbound and eluted fractions were added with 0.05% NaN_3_ and kept at 4°C.

### Antibody concentration, medium change and separation of IgA from IgM by gel filtration chromatography

Immunoglobulin fractions containing IgG and IgA-IgM antibodies were concentrated on Amicon cartridges; the latter ones were charged on the Bio-Silect SEC 400–5 column (BioRad) to separe IgA from IgM antibodies. Columns were extensively washed with water and equilibrated with sterile PBS buffer (7 volumes; flow: 200 microL/min). Samples were also applied and eluted in PBS buffer (flow: 1 mL/min), and fractions containing IgA were collected and sterilized onto 0.22 micron membranes before storage at −80°C. Columns were extensively washed with sterile water (7 volumes; flow: 200 microL/min), added with 0.05% NaN_3_ and stored at 4°C.

### Affinity purification of mucosal IgA1 on jacalin-agarose

Affinity purification of IgA1 fractions from mucosal fluids was obtained by chromatography on jacalin-bound agarose 4% beads (SIGMA-ALDRICH), according with a protocol previously described [25]. Jacalin is a lectin protein purified from *Artocarpus integrifolia*, able to bind D-galactose; this sugar can be specifically found in glycans on IgA1, but not on IgA2 molecules.

In detail, 3–5 mL of genital fluids, diluted in phosphate buffer 1x, were applied on 2 mL of jacalin-agarose resin, packed in Bio-Scale MT2 column (BioRad), at 150 microL/min. After extensive washing, IgA1 fractions were eluted with D-galactose 0.8 M dissolved in sterile water, at 200 microL/min. Eluted antibodies were concentrated with Amicon ultra-centrifuge filter devices (Millipore), resuspended in a small volume of PBS 1× and sterilized on 0.22 µm membranes before storage at −80°C.

### Statistical analysis

In order to identify the condition assuring the highest immunoglobulin recovery, two separated linear regression models for repeated measurement - one for IgA and one for IgG (response variable) - were fitted to compare different levels of concentration under different experimental conditions. Since the response variable was not normally distributed, different transformations of data (logarithmic, square root, etc.) were tested, by the QQplot, a graphic which relates the empirical quantiles and the quantiles of a standard normal distribution [26]. A log-transformation of the response variable was chosen. The regression model chosen took into account different types of correlation between measurement between-subject, through the specification of a particular correlation matrix. In this study, a compound symmetry correlation matrix was chosen.

The initial model, including all variables of interest (such as experimental condition, presence of HIV infection, incubation and interaction), was fitted. The significance of each variable was evaluated by means of F-test. A backward stepwise procedure was applied, to select the variables to be included in the final model. In order to avoid false positive differences between the means of concentration determined under different experimental conditions, a Tukey adjustment for paired comparison was used [27]. All tests were two-tailed and were considered statistically significant if the p-value associated was <0.005. Data were analyzed using the proc mixed of SAS Version 9.1 (SAS Institute, Cary, NC, USA).

## Results

In the first phase of this study, optimal conditions to collect and preserve genital fluids, to isolate and to quantify immunoglobulins, particularly IgA, were set up. Optimal assay conditions were determined through the analysis of a cohort of male and female Italian people, including healthy and HIV-positive individuals (n = 89).

**Figure 1:**
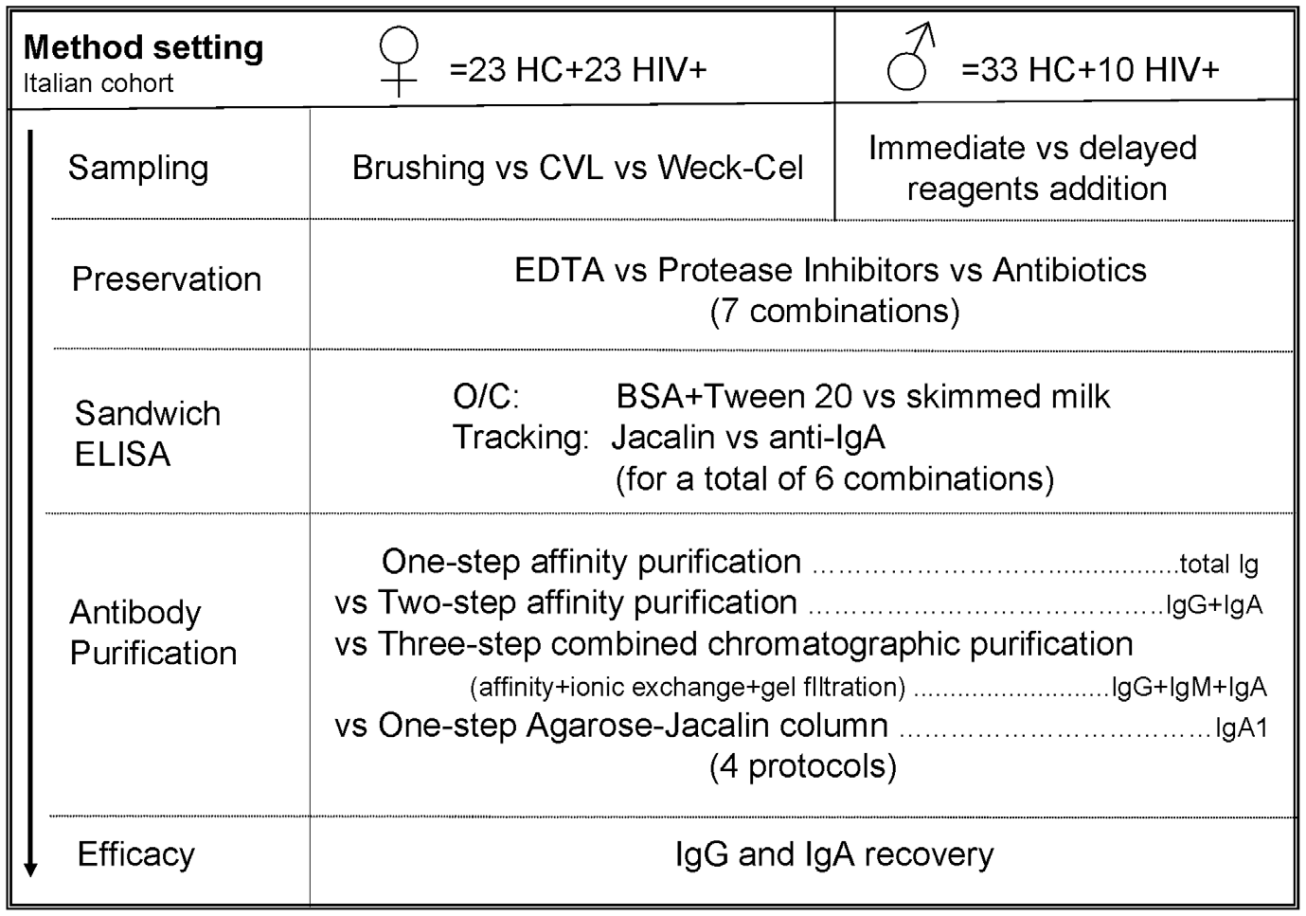
Design of the study. The scheme summarizes the methods tested in each step of immunoglobulins purification and quantitation.

In the second phase, methods and experimental conditions were assayed on a cohort of Cambodian women (n = 20), both HIV-positive and healthy subjects, in order to confirm sensitivity and reliability of results on a population characterized by a different genetic background and possibly by a different immunologic responsiveness to HIV. **Figure 1** summarizes the design of the whole study.

### Fluid sampling and processing from female individuals

#### Comparison: Sampling methods

A group of 23 healthy and a similar number of HIV-positive women underwent extensive vaginal sampling during routine gynecological examinations. In order to minimize physiological variation in immunoglobulins concentration, naturally occurring during menstrual cycle and to improve antibody recovery [22], sampling was carried out in the post-ovulatory phase, i.e. 15–20 days after the first day of the latest cycle. The use of PBS buffer at neutral pH values (pH 7.2–7.4), although different from the vaginal physiologic pH (pH 5.5), was not found to affect immunoglobulin recovery significantly. The optimal volume of CVL buffer to be used was determined by a series of preliminary sampling which employed variable volumes of buffer (3–10 mL), according with methods previously published [20]; 7 mL was the optimal volume that allowed a good antibody recovery and prevented either excessive viscosity or unnecessary dilution of the sample.

Standard sandwich ELISA assays showed that immunoglobulin recovery was greater with CVL method than with brushing, due to the better opportunity to reach and deterge the whole mucosal surface. IgG recovery obtained with CVL sampling method ranged between 10–43 microg/mL, and IgA were found between 5–21 microg/mL. Brushing gave undetectable IgG and IgA concentrations in a high percentage of samples (10/13 samples  = 76.9%); in other words, immunoglobulin concentrations were lower than 0.125 microg/mL, that was the lower limit of assay detection.

Weck-Cel was superior to CVL method, due to the complete recovery of mucosal antibodies that was achieved; these results were noteworthy, because the buffer volume recovered by CVL was by far larger than that recovered by sponges (7 vs. 0.05 mL respectively).

Indeed, immunoglobulin concentrations obtained with sponges ranged between 5–128 microg/mL for IgG and between 3–58 microg/mL for IgA, in agreement with previous observations [15]. IgA and IgG concentrations found in each specimen were found concordant, thus confirming that Weck-Cel sampling methods do not introduce systematic bias in concentrations of a single immunoglobulin type.

#### Comparison: Mucosal fluid preservation

Normal vaginal bacteria, mucus and lytic enzymes from cell debris are physiologic components of genital fluids, but they can influence antibody recovery and evaluation. The presence of spermatozoa was also checked by optical microscopy, although participants were asked to avoid intercourses 24–48 hours before sampling, specimens were controlled. However, male cells, when present, did not affect antibody recovery and/or purification significantly (data not shown).

Genital fluids from healthy women (HC) and from HIV-positive patients were processed within 30 min from collection. Each individual sample was split in multiple aliquots (brushing, CVL), or was collected in multiple replicates (Weck-cel); one aliquot for each individual sample was respectively added with:

- cryopreservation only
- EDTA
- Protease inhibitor mix (IP)
- antibiotic mix (AB)
- EDTA + protease inhibitor mix (IP)
- EDTA + antibiotic mix (AB)
- protease inhibitor mix (IP) + antibiotic mix (AB)
- EDTA + antibiotic mix + protease inhibitor mix.

Sample freezing and thawing was kept at minimum, and samples stored for more than three months were not evaluated in the study. In this condition, no significant association between length of storage and sampling conditions was found. However, IgG were more sensitive to freezing/thawing, while IgA were not significantly affected by the event.

In order to assess the importance of quick processing, experiments were carried out on a panel of specimens rapidly processed (indicated as “immediate” mode) and on a series kept overnight at 4°C before processing (indicated as “delayed” mode).

IgG concentrations from HC were at least one order of magnitude higher than IgA ones, and ranged between 241 and 874.3 microg/mL in the “immediate” mode and between 221 and 682 microg/mL in the “delayed” mode.

IgA concentrations ranged between 214.6 and 565.6 microg/mL (“immediate” processing mode) and between 192.7 and 530.6 microg/mL in “delayed” mode. HIV-positive vaginal fluids showed a lower concentration of IgG and of IgA antibodies, probably due to immunocomplexes with viral proteins (range: IgG 208–678 microg/mL; IgA 18–788 microg/mL). IgA detectability was never lost, although few individual samples showed an occasional reduction of IgA values.

The effect of additives was compared in a statistical model which evaluated all experimental parameters, as described in Methods. Some variables, such as the HIV serostatus, were excluded from the final model, because they were not influential. Similarly, all interactions between variables, such as that between the “type of additive” and the “processing mode”, were excluded from the final model. As shown in **Table 1**, the statistical model took into evaluation the cumulative panel of data (“joint”) as well as the hypothesis of the two comparable “immediate” and “delayed” processing modes.

**Table 1:** Statistical analysis of Modes and Additives in immunoglobulin recovery from vaginal fluids.

Mode	Additive	IgG			IgA		
		*vs*	Basic	Adj	*vs*	Basic	Adj
Jo	Null	---	---	---	---	---	---
	EDTA	---	---	---	---	---	---
	IP	---	---	---	---	---	---
	AB	Null	0.0509	---	Null	0.0061	---
	EDTA+IP	Null	0.0336	---	---	---	---
		EDTA	0.0016	0.0344	---	---	---
		IP	0.0011	0.023	---	---	---
		AB	0.001	0.0026	---	---	---
	EDTA+AB	EDTA+IP	0.0084	---	Null	0.001	0.0221
		---	---	---	EDTA	0.0363	---
		---	---	---	IP	0.028	---
		---	---	---	EDTA+IP	0.015	---
	IP+AB	Null	0.022	---	EDTA+AB	0.0381	---
		EDTA+IP	<0.0001	0.0007	---	---	---
	EDTA+ IP +AB	EDTA+IP	0.0005	0.0111	---	---	---
I	Null	---	---	---	---	---	---
	EDTA	Null	0.0327	---	---	---	---
	IP	---	---	---	---	---	---
	AB	Null	0.0243	---	Null	0.034	---
					IP	0.0299	---
	EDTA+IP	EDTA	0.0003	0.0061	---	---	---
		IP	0.0073	---	---	---	---
		AB	0.0002	0.004	---	---	---
	EDTA+AB	EDTA+IP	0.0064	---	---	---	---
	IP+AB	---	---	---	---	---	---
	EDTA+ IP +AB	EDTA+IP	0.0015	0.0316	---	---	---
D	Null	---	---	---	---	---	---
	EDTA	---	---	---	---	---	---
	IP	---	---	---	---	---	---
	AB	---	---	---	---	---	---
	EDTA+IP	IP	0.0393	---	---	---	---
	EDTA+AB	EDTA+IP	0.001	0.021	EDTA+IP	0.0326	---
	IP+AB	Null	0.0242	---	---	---	---
		EDTA	0.0173	---	---	---	---
		EDTA+AB	0.0219	---	---	---	---
	EDTA+ IP +AB	---	---	---	Null	0.0403	---

Modes: Jo, joint; I, Immediate; D, delayed. Additives legends: Null, cryopreservation only; EDTA: ethylenediaminetetraacetic acid; IP, protease inhibitors; AB, Antibiotics. Only significant P values (before and after adjustment, Basic and Adj, respectively) were included in the Table.

According to IgG-specific statistical modeling, the use of two additives (i.e. EDTA+IP or EDTA+AB or IP+AB) significantly improved IgG recovery in respect to the use of single additives; and so did the use of the triple association EDTA+AB+IP. The double IP+AB component and the triple additive retained their significativity after model adjustment, when analyzed in the “joint” model.

In the IgA statistical modeling, addition of AB was significant both as unique component and in addition with a second agent (AB+EDTA or AB+IP). The triple additive was significantly superior to other protocols in the “delayed” mode only. No additive retained its significativity in the adjusted model.

IgG recovery was significantly associated with the type of additive used (F = 2.27; Probability >F, 0.0306). Other variables taken in consideration in the statistical model, such as the mode of treatment (i.e. pre- or post-incubation addition of preserving agents) or the HIV status (i.e. HIV-positive or -negative) were not influential. Interaction between experimental variables, namely between the processing mode and the type of additives, was excluded, thus confirming the significativity of the model (F = 3.72; Probability >F, 0.0008).

IgA recovery was significantly associated with the processing mode but not with the type of agents employed (F = 19.4; Probability >F <0.0001). When interaction between “mode” and “treatment” variables was excluded, IgA concentrations resulted significantly associated with both the single variables (mode: F = 18.65; Probability >F <0.0001; treatment: F = 2.29; Probability >F <0.0280).

According with experimental observations and with statistical analysis, the “immediate” processing, albeit not significant, led to higher IgG and IgA recovery, and was therefore considered more suitable than the “delayed” one. Two different additives enhanced IgG and IgA recovery, i.e. IP+AB and EDTA+AB, respectively. The optimal procedure therefore consisted in applying two separate procedures for IgG and IgA to each vaginal sample to be processed.

### Fluid sampling and processing from male individuals

Normal seminal fluid is highly viscous and can not be used directly to isolate mucosal antibodies. Moreover, it contains endogenous lytic enzymes and cell debris that may affect antibody recovery and evaluation. Due to its features, male fluid requires a pre-incubation (4°C for 1 h), a proper dilution in sterile PBS, the separation of spermatozoa by centrifugation (1,800 g for 10 min). Finally, it also needs inactivation of complement proteins (57°C for 30 min), a procedure which enhances antibody testing and inactivate HIV particles, if present. Addition of protease inhibitors is also mandatory, to prevent antibody loss due to enzymatic lysis. Protocols here described did consider optimal composition and timing for preserving additives in comparison with a published method [23].

Optimal sampling and storage conditions were determined by comparing seven additives in two main modes of addition (“immediate” vs “delayed”) and their controls, for a total of 16 aliquots. In the “immediate” mode, the seven agents (indicated below as b–h) were added to the fresh whole seminal fluid before any further processing, while in the “delayed” mode, component addition was postponed (nine samples). Control samples were kept untreated (a) or treated according with a published method (i, or “Mestecky” method)

- cryopreservation only (negative control)
- EDTA
- Protease inhibitor mix (IP)
- antibiotic mix (AB)
- EDTA + protease inhibitor mix (IP)
- EDTA + antibiotic mix (AB)
- protease inhibitor mix (IP) + antibiotic mix (AB)
- EDTA + protease inhibitor mix + antibiotic mix
- Positive control (Mestecky method)

#### Comparison: seminal fluid processing

Antibody recoveries ranged from 506.3 to 820.4 microg/mL for IgG and from 239.63 to 455.73 microg/mL for IgA in samples processed by “immediate” mode and from 436 to 665.9 microg/mL for IgG and from 210.54 to 418.96 microg/mL for IgA in samples undergone to the “delayed” mode.

Both modes were superior to the simple cryoconservation (indicated as the “null” additive), that was the negative control of the experiment. Higher IgG recoveries were achieved in the “immediate” mode, when components were immediately added to seminal fluids. The “immediate” IgG recoveries were also superior to those obtained with the positive control protocol (Mestecky method). Similarly, IgA recoveries were higher in “immediate” samples.

**Figure 2:**
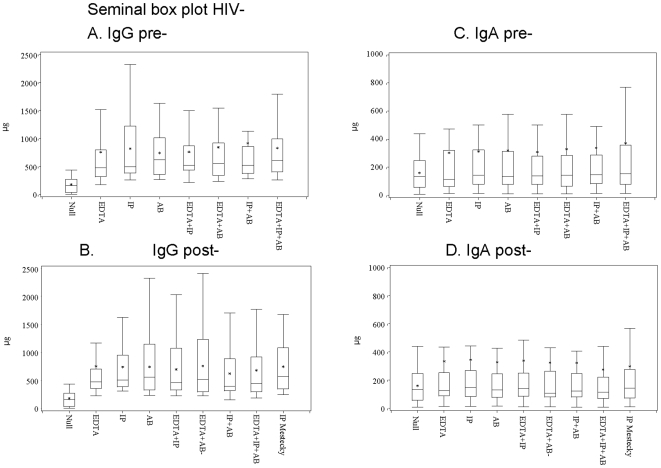
Immunoglobulin concentration in seminal fluids from Italian men. Box plot summarizes the immunoglobulin concentrations (median and range) observed in seminal fluids from healthy donors, according to the Additives (cryopreservations only, single, double or triple additives) and the Processing Modes (addition of preserving agents made pre- or post-fluid incubation). All specimens were immediately processed after sampling; the values are given in microgram/mL. Panel legends: A. IgG range in pre-incubation samples; B. IgG range in post-incubation samples; C. IgA range in pre-incubation samples; D. IgA range in post-incubation samples. Additives legends: Null, cryopreservation only; EDTA: ethylenediaminetetraacetic acid; IP, protease inhibitors; AB, Antibiotics. Box legends: Box short sides represent the third (Q3) and the first quartile (Q1), respectively, while the asterisk (*) within the box indicates the mean and the horizontal bar (--) shows the median value, respectively. Vertical lines above or below the box indicate the corresponding quartile value (Q3 or Q1) plus or minus 1.5 times the interquartile interval (IQ = Q3−Q1).

Comparison of median values and of maximal immunoglobulin concentrations, summarized in **Table 2** and in **Figure 2**, showed that addition of IP alone or of the triple additive achieved the higher IgG recovery, as shown by the higher median and maximal values observed in the “immediate” mode. Similar considerations could be drawn for IgA recovery, which was maximal when the triple additive was used.

**Table 2:** Statistical analysis of Modes and Additives in immunoglobulin recovery from seminal fluids.

Mode	Additive	IgG			IgA		
		*vs*	Basic	Adj	*vs*	Basic	Adj
I	Null	---	---	*	Null	<0.0001	*
	EDTA	Null	<0.0001	*	Null	<0.0001	*
	IP	Null	<0.0001	*	Null	<0.0001	*
	AB	Null	<0.0001	*	Null	<0.0001	*
	EDTA+IP	Null	<0.0001	*	Null	<0.0001	*
	EDTA+AB	Null	<0.0001	*	Null	<0.0001	*
	IP+AB	Null	<0.0001	*	Null	<0.0001	*
	---	EDTA	0.0463	*	---	---	---
	---	AB	0.0005	*	---	---	---
	EDTA+ IP +AB	Null	<0.0001	*	Null	<0.0001	*
	---	---	---	---	EDTA	0.0043	*
	---	---	---	---	IP	0.0041	*
	---	AB	0.0236	*	AB	0.0163	*
	---	---	---	---	EDTA+IP	0.0284	*
D	Null	---	---	---	---	---	---
	EDTA	Null	<0.0001	*	Null	<0.0001	*
	IP	Null	<0.0001	*	Null	<0.0001	*
		---	---	---	EDTA	0.1560	---
	AB	Null	<0.0001	*	Null	<0.0001	*
		---	---	---	IP	0.0131	*
	EDTA+IP	Null	<0.0001	*	Null	<0.0001	*
	EDTA+AB	Null	<0.0001	*	Null	<0.0001	*
		---	---	---	IP	0.0367	*
	IP+AB	Null	<0.0001	*	Null	<0.0001	*
		---	---	---	IP	0.0118	*
	EDTA+IP+AB	Null	<0.0001	*	Null	<0.0001	*
		EDTA	0.0373	*	EDTA	0.0005	*
		IP	0.0016	*	IP	<0.0001	*
		AB	0.0265	*	AB	0.0009	*
		---	---	---	EDTA+IP	0.0001	*
		EDTA+AB	0.0072	*	EDTA+AB	0.0061	*
		---	---	---	IP+AB	0.0017	*
	Mestecky	Null	<0.0001	*	Null	<0.0001	*

Modes: I, immediate; D, delayed. Basic, basic model; Adj, adjusted values. Additives legends: Null, cryopreservation only; EDTA: ethylenediaminetetraacetic acid; IP, protease inhibitors; AB, Antibiotics. Only significant P values were included in the Table; asterisks indicated P values maintaining their significativity after model adjustment (Adj).

The comparison of seminal fluids from healthy donors with those from HIV-positive cohort showed that HIV serostatus did not affect antibody recovery significantly, therefore, this variable was excluded from the statistical model.

According with statistical modeling, presented in **Table 2**, all additives obtained significant differences (p<0.05) in respect to negative control (cryoconservation only, indicated as “null”); some additives or their associations were also found significantly superior to others. In detail, the association IP+AB was found significantly superior to addition of single components for IgG (“immediate” mode), and for IgA (“delayed” mode). Similar results were observed with the triple association EDTA+IP+AB, which was found superior to all single components and to at least two out of three double additives. The positive control method was uniquely superior to negative control, but was not found superior to any of the other additive tested.

Both the experimental observations (mean Ig values), shown in **Figure 2**, and the statistical analysis confirmed that “immediate” addition of IP+AB was the method more suitable to enhance IgG recovery (mean value: 919.85 total µg), while the triple combination (EDTA+AB+IP) provided higher amounts of IgA (mean value: 371.36 total µg). These two additives were therefore used in sample processing before setting of methods for antibody purification.

### Setting of sandwich ELISA assay

Female and male genital fluids were used to set up the optimal protocol of a IgA-specific sandwich ELISA assay. A sandwich ELISA method suitable to detect mucosal IgG and especially IgA needs high specificity and sensitivity, in order to ignore contaminant within genital fluids and to reveal also low IgA concentrations.

**Figure 3:**
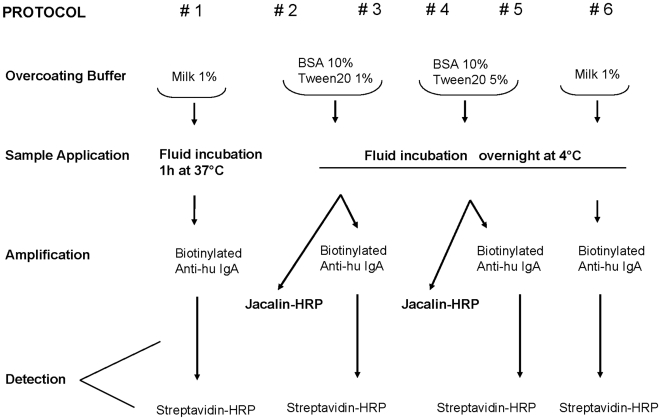
Sandwich ELISA assay. The scheme introduces the six sandwich ELISA protocols that were compared in the study.

Six different protocols were compared in the study; they were different in coating conditions, in immunoglobulin detection and in signal amplification, as summarized in **Figure 3**. Jacalin was not used in the protocol #6 because of its binding to milk, that was likely to reduce test specificity. All protocols were compared with the protocol #1, which was the standard method to detect serum IgA. Standard dilutions of human IgA (1∶1-1∶32), ranging from 4 mg/mL to 0.125 microg/mL, were included in the analyses and compared with similar dilutions of mucosal fluids.

The intra-assay variability was standardized by performing two replicates for each sample dilution; each value was compared to the standard curve. The inter-assay variability was controlled by standardizing the experimental values on the reference IgG and IgA curves; the reference values were compared in each ELISA plate.

#### Detection Specificity

In order to assess specificity of commercial reagents used to detect IgA and IgG in the sandwich ELISA assay, a preliminary experiment of cross-detection was performed in two replicas. Standard curves of IgA and IgG dilutions (1∶1-1∶10^5^) ranging from 4 microg/mL to 40 picog/mL were revealed in sandwich ELISA tests using either anti-human-IgA or anti-human-IgG conjugated antibodies. Moreover, antibodies from two different providers were compared in the experiment (DAKO, Glostrup, Denmark, and Southern Biotech, SB, Birmingham AL, USA); finally we chose reagents from SB, due to their lower background and the higher analytical reproducibility. Anti-immunoglobulins from other providers were not assayed in the study. Results, summarized in **Table 3**, showed that IgG-IgA cross-reactivity was uniquely observed at high concentration of standard antibodies (i.e. at the first two dilution points of the dilution scale).

**Table 3:** Cross-reactivity of commercial anti-human IgA and IgG reagents used in sandwich ELISA assays.

Anti Human serum #1	IgA						IgG					
(from DAKO)	1	1.10	1∶100	1∶1,000	1∶10,000	1∶100,000	1	1.10	1∶100	1∶1,000	1∶10,000	1∶100,000
**Anti-IgA**	2,049±	1,903±	1,733±	646±	154±	67±	**1,450±**	**494±**	133±	37±	26±	29±
	32	42	48	37	28	20	**45**	**39**	22	21	26	24
**Anti-IgG**	**815±**	**304±**	101±	53±	23±	15±	1,939±	1,991±	1,965±	1,701±	918±	420±
	**50**	**45**	25	28	24	21	48	43	37	40	34	47
**Anti Human serum #2**	**IgA**						**IgG**					
(from Southern Biotech)	1	1.10	1∶100	1∶1,000	1∶10,000	1∶100,000	1	1.10	1∶100	1∶1,000	1∶10,000	1∶100,000
**Anti-IgA**	1,947±	1,946±	1,572±	627±	164±	90±	**1,449±**	**469±**	120±	37±	21±	33±
	53	48	46	49	36	20	**42**	**47**	35	31	24	27
**Anti-IgG**	**809±**	**301±**	105±	40±	20±	10±	1,925±	1,998±	1,893±	1,704±	966±	467±
	**45**	**43**	38	30	28	27	46	55	51	48	40	32

Dilutions: 1∶1 = 4 microg/mL; 1∶10 = 400 ng/mL; 1∶10^2^ = 40 ng/mL; 1∶10^3^ = 4 ng/mL; 1∶10^4^ = 400 pg/mL; 1∶10^5^ = 40 pg/mL. Response variability was also reported in the Table.

Results are expressed in OD values.

#### Comparison: Sandwich ELISA Specificity and Sensitivity

The six sandwich ELISA protocols to be tested were compared on different aliquots from 18 genital fluids from healthy donors and on an equal number of samples from HIV-positive people (females, n = 13; males, n = 5 in both groups). Each aliquot underwent a different protocol, so that the genital fluids from each donor were analyzed in all conditions. In most protocols, IgA resulted undetectable or the assay background prevented the evaluation of results. In protocol #1, the mean IgA concentrations from female fluids were 103.4 microg/mL (range: 6–250 microg/mL) in healthy people and 219.3 microg/mL (range: 4–780 microg/mL) in the HIV-positive cohort; protocol #6 even achieved higher IgA mean values, that were 297.1 microg/mL (range: 16.6–687 microg/mL) in the healthy cohort and 682.9 microg/mL (range: 11.1–2692.1 microg/mL), in the HIV-positive group, respectively. High IgA concentrations from seminal fluids were also obtained with protocol #6, with IgA mean values, 353.02 microg/mL (range: 21.6–453 microg/mL) in the healthy cohort and 582.9 microg/mL (range: 25.2–1192.2 microg/mL), in HIV-positive group, respectively. The two populations (healthy and HIV positive subjects) did not show significant differences in antibody concentrations.

Protocols employing BSA in coating buffer (#2-#5) showed higher aspecific binding than the milk-based protocols (#1 and #6); The increase in the concentration of Tween 20 detergent (1% vs 5%), evaluated in the protocols #2-#4 and #3-#5, respectively, was unable to control test aspecificity. Skimmed milk indeed enhanced the specific binding of antibodies, as shown by the comparison of protocols #1 (the control method) and #6.

Jacalin-HRP detection (protocols #2 and #4) provided a lower sensitivity than the use of conjugated anti-human immunoglobulins and biotin-streptavidin amplification (protocols #1, #3, #5 and #6). Conversely, the increased timing of antibody capture (from one hour to overnight) enhanced test sensitivity.

In conclusion, the combination of reagents and parameters set in the protocol #6 provided the higher specificity and sensitivity among the conditions evaluated, and it also offered a significant improvement of the standard method (#1) to detect IgA in genital fluids.

### Immunoglobulin recovery

**Figure 4:**
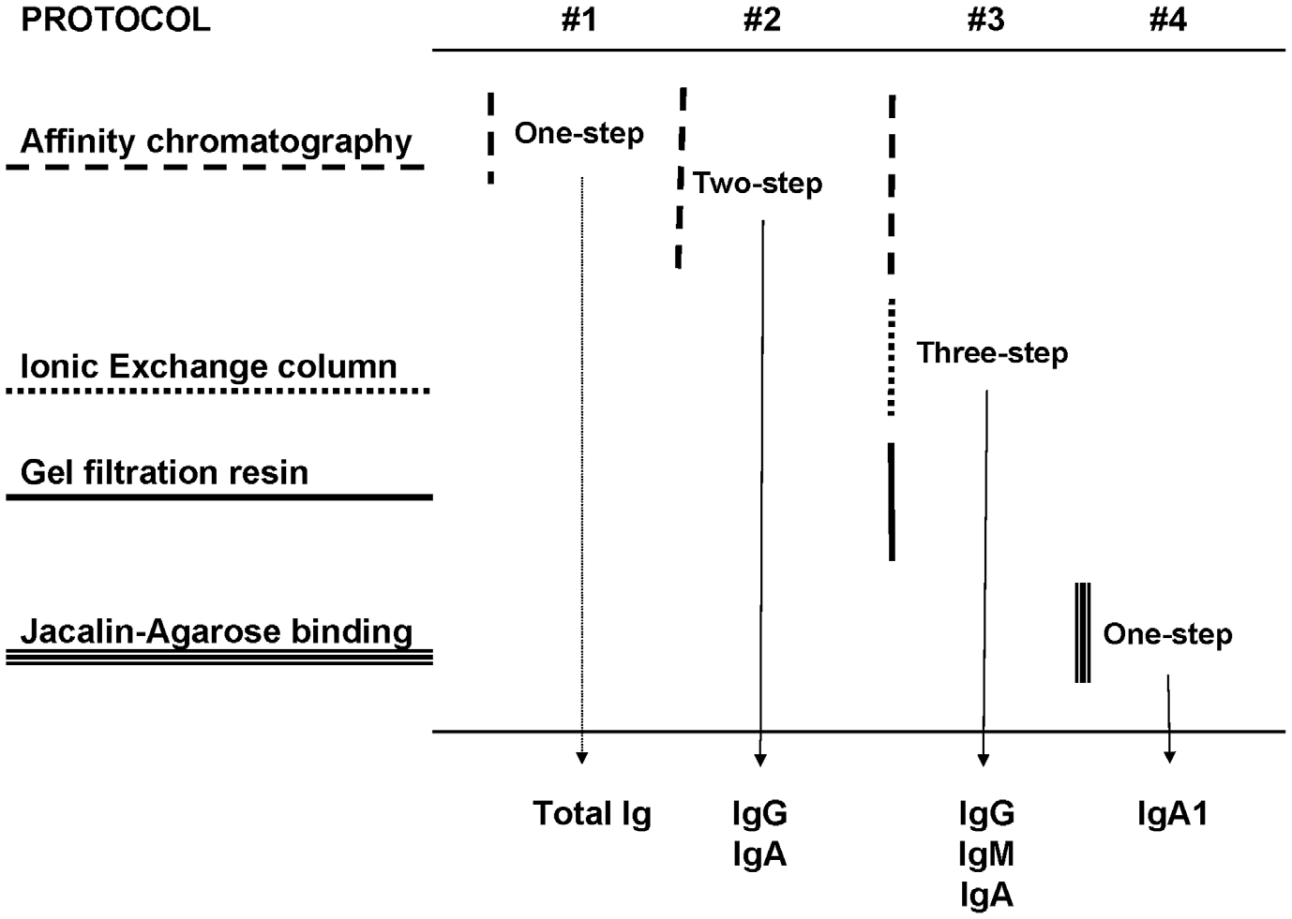
Affinity purification of immunoglobulins. Comparison of the four chromatographic methods for immunoglobulin isolation that were evaluated in the study.

Four protocols for IgG and IgA purification were compared in the study. As summarized in **Figure 4**, methods consisted in the one-step, total IgG-IgA fractioning on affinity sepharose columns; the two-step, IgG and IgA separation on sequential columns; the three-step, IgG affinity separation, followed by IgA isolation by ionic exchange plus gel filtration; the IgA1 affinity separation on a jacalin-agarose column. Assays were carried out on a panel of aliquots from ten female and seven male genital fluids. In this way, fluid from each donor underwent all purification protocols under assay.

In order to rule out any systematic method bias that could limit IgA recovery, standard human IgA and IgG were mixed at concentrations similar to those observed in normal serum and applied on columns. Both standard IgA and IgG from the mock sample were fully recovered and no residual immunoglobulins were detected in elution buffer.

Due to the peculiar composition of genital fluids, various interfering components, such as mucin and glycoproteins, were likely to interfere with column purification. The hypothesis of interference was addressed by purifying both sera and genital fluids from two individuals under the same experimental conditions. Serum IgA were higher than the mucosal counterparts; moreover, several small-volume elution cycles were required to recover mucosal antibodies, a confirm of the fact that the affinity purification of mucosal antibodies requires peculiar conditions to be accomplished.

Neither interfering proteins or glycosylated moieties affected male and female fluid purification. No further adjustments or supplementary steps were added to the protocols to process male and female genital fluids; finally, statistical modeling was not required to compare the four sets of results.

#### One-Step, total Immunoglobulin affinity purification

The assay employed sepharose affinity columns carrying commercial antibodies recognizing human IgG, IgA and IgM, to set up optimal conditions to capture total Ig from genital fluid specimens. Immunoglobulin-conjugated sepharose (5 mL) completely retained antibodies from 20–100 microL of vaginal or seminal fluids; however, the efficiency of elution from the resin and the antibody yield were not reproducible in different purification sessions (range: 107–328 microg of total Ig per mL of sample). IgA fraction was poorly eluted from total Ig fractions. Moreover, the capturing anti-human antibodies were detached from the resin following repeated cycles of elution: this was a control procedure carried out on columns devoid of sample. Most importantly, detached capture antibodies reached non-negligible concentrations, that were higher than the cut-off values stated for the quantitative ELISA assay. Due to these drawbacks, the one-step purification of total immunoglobulins was considered unfeasible to obtain mucosal antibodies.

#### Two-step, IgG- and IgA-specific affinity purification

Two-step purification consisted in a tandem IgA-IgG separation, done by loading genital fluids on IgA-capturing and subsequently on IgG-binding sepharose columns. As observed in the one-step method, 100 microL was the optimal fluid volume to be loaded onto 2.4 mL columns both for female and male specimens. Immunoglobulins yields ranged between 135–1,444 microg of IgG per mL of sample and <0.125–125 microg of IgA per mL of sample, respectively. The proportions of recovered IgA were lower than those of IgG antibodies, a finding that could be related to the higher IgG concentration observed in genital fluids. Furthermore, IgA recovery was not reproducible in different sessions, even when aliquots from the same mucosal specimen were purified. Different experiments were performed to overcome this limitation and set upthe method conveniently.

In order to rule out that under-reactivity or cross-reactivity might have affected ELISA detection, both eluted and antibody-void fractions underwent ELISA testing and were detected with both anti-IgA and anti-IgG antibodies. The anti-IgA reagent failed in detecting IgG antibodies purified from the column, therefore showing no reagent cross-reactivity and no accidental IgA presence. Conversely, the anti-IgG antibodies showed a weak cross-reactivity to eluted IgA. As expected, the anti-IgA reagent was not reactive with antibody-void volumes from IgG column (presumably devoid of IgA); unexpectedly, the anti-IgG reagent failed in detecting the IgA-void volume from IgA column, which was still containing IgG. This lack of reactivity was not due to IgG shortage in the fluid, because this material regularly yielded IgG, once loaded on the IgG-specific column. Moreover, all mucosal fluids assayed, but not sera tested as controls, shared this effect; it was finally attributed to an interfering component, presently undetermined, that was uniquely present in mucosal fluids.

Due to the multiple drawbacks that were encountered in all phases of the two-step affinity purification, this method was considered unsuitable to isolate mucosal immunoglobulins.

#### Three-step IgG and IgA affinity column purification

**Figure 5:**
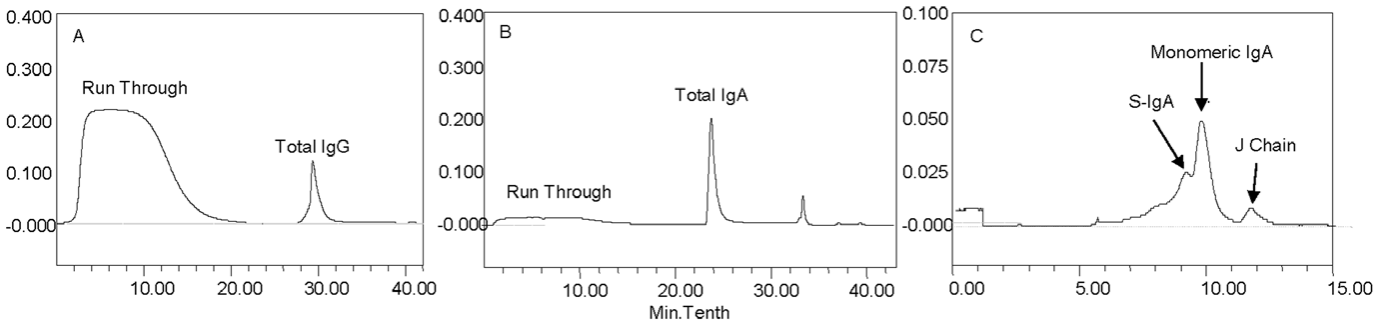
Immunoglobulins purification from vaginal fluids. Panel legends: A. IgG purification by Protein G column. B. IgA purification by ionic exchange column. C. IgA subtypes isolation by gel filtration.

In the three-step purification method, genital fluids were first loaded on sepharose-Protein G columns, in order to obtain IgG fractions. Subsequently, unbound IgM and IgA were recovered as a whole fraction, on a resin column for anionic-exchange, after the second step of the method. After the concentration of the fraction, the gel filtration on Bio-Silect columns led to isolate IgA from IgM. **Figure 5** shows three representative chromatograms, where recovery of IgA subfractions is reported. In the three-step method, 3–5 mL of female or male fluids were the optimal volumes to be loaded onto 1 mL columns. Despite the labour required to accomplish all phases of the protocol, IgG and IgA yields were higher than those obtained by the other protocols previously assayed. Immunoglobulin ranges were, respectively: IgG, 100–1,400 microg/mL of sample (after Protein-G chromatography); IgA, 11.2–438 microg/mL of sample (after anionic exchange chromatography); after gel filtration chromatography, IgA ranges were similar to the previous ones, with a reduction of 10%. Immunoglobulin recovery was not affected by immuno-complexes, because the first purification step, performed in Glycine buffer pH 2.0, prevented their formation. Notably, the three-step purification yielded more reproducible results than the other methods tested, and therefore it was chosen to isolate immunoglobulins in the study.

#### One-step IgA1 Jacalin-agarose affinity purification

Jacalin weights 54/65 kDa and contains four identical subunits, able to bind D-Galactose belonging to O-linked carbohydrate chains found on IgA1 and on other glycoproteins. Due to its properties, jacalin has been used to purify mucosal immunoglobulins since 1987 [25].

Agarose-bound jacalin specifically captures monomeric, dimeric and secretory IgA1 molecules, and can separate these molecules from IgA2, IgG, IgM, IgD, IgE and from the secretory component (SC), either free or bound to J chain. IgA1 is an immunoglobulin subtype that is specifically elicited in response to viruses, while the IgA2 subtype takes part in the antibacterial response. Moreover, IgA1 antibodies are largely predominant in female genital fluids during the post-ovulatory period, while IgA2 are negligible or even absent [16].

After elution from the agarose-jacalin column, the rates of antibody recovery were determined by sandwich ELISA and compared with antibody concentrations determined in IgA-void volumes. Jacalin purification was very specific, due to the weak OD values observed when ELISA assays were developed with anti-IgG antibody; these OD values fell within the cut-off values at second/third sample dilution. Previous experiment already showed that the anti-human IgG reagents used in the ELISA assay cross-reacted with IgA immunoglobulins found in eluted buffers (see the “ELISA assay” section for details). As a further confirmation of jacalin specificity, high concentrations of IgG were found in IgA-void volumes, because mucosal IgG were not bound by the lectin.

The incubation temperature did not affect the immunoglobulin binding to jacalin significantly, while prolonged incubation intervals (i.e. overnight contact) increased the immunoglobulin yield from female genital fluids; this result, observed both in HIV-positive and healthy specimens, was confirmed by ELISA assays performed on void volumes.

The anti-IgA reagents used to develop the ELISA assays cross-reacted with IgG found in IgA-void volumes (see the “ELISA assay” section for further details). This anti-IgA reactivity to IgA-void volumes was not due to residual IgA2 immunoglobulins: as mentioned above, this antibody subtype is rare, if any, in post-ovulatory vaginal specimens, and can not be bound by jacalin. Indeed, the observed reactivity was due to IgG; when IgA-void volumes from the jacalin column underwent a purification cycle on the IgG-specific column, both anti-IgG and anti-IgA signals on the resulting immunoglobulin-void volumes were abolished.

#### Comparison: antibody affinity purification vs ionic exchange vs jacalin chromatography

Parallel, comparative experiments of genital fluid affinity purification on sepharose-anti-IgA and on jacalin-agarose columns were performed on a panel of 20 genital fluids, obtained from ten healthy women and ten HIV-positive patients.

**Figure 6:**
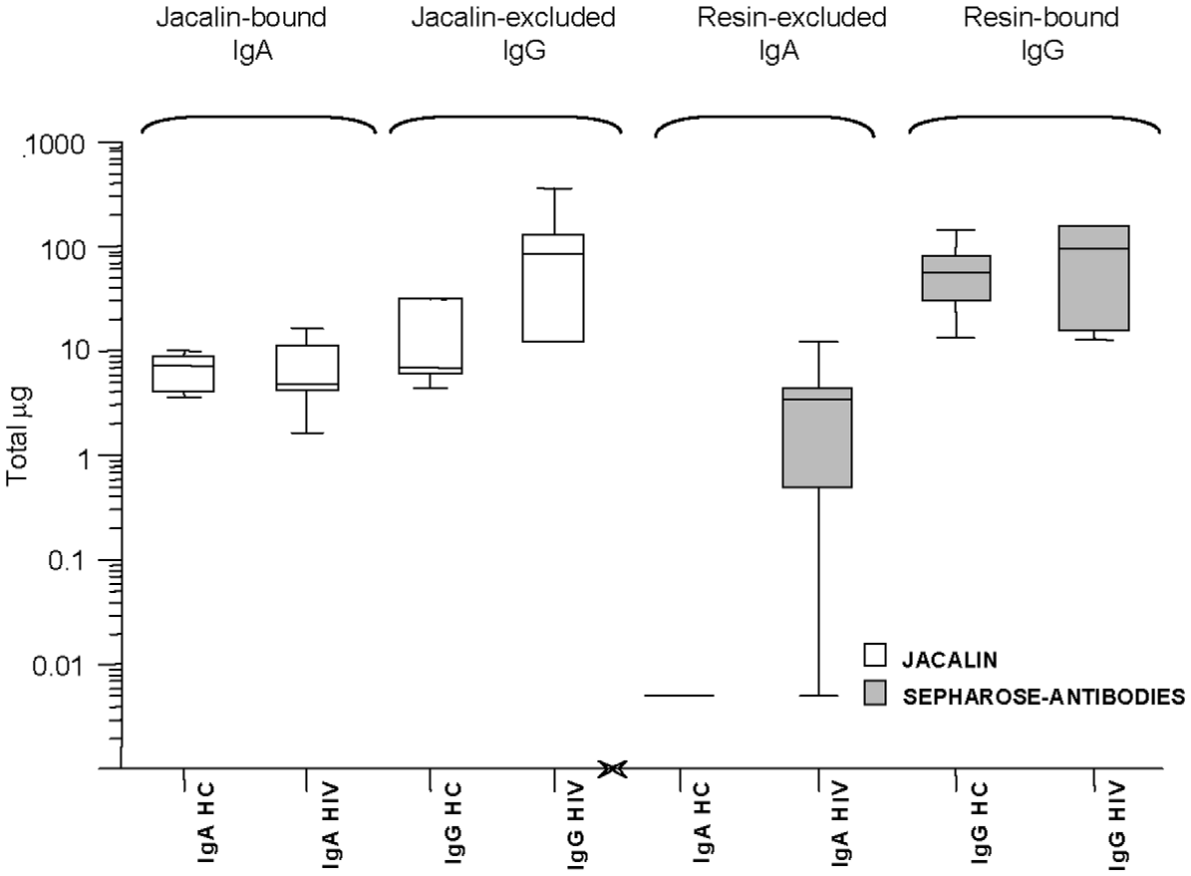
Three-step vs jacalin affinity purification of mucosal immunoglobulins. Box plot comparing IgA and IgG immunoglobulin purification by two different methods assayed in the study. Left panel shows IgA and IgG values measured in jacalin-bound and -excluded fractions, respectively; right panel presents IgA and IgG values measured in the two bound fractions. Values are expressed in total micrograms per sample, in log-scale. From the left to the right, respectively: Jacalin–agarose purification (white box): IgA from healthy controls; IgA from HIV-positive subjects; IgG from healthy controls; IgG from HIV-positive subjects. Three-step affinity purification (grey box): IgA from healthy controls; IgA from HIV-positive subjects; IgG from healthy controls; IgG from HIV-positive subjects. Box legends: Box short sides represent the third (Q3) and the first quartile (Q1), respectively, while the horizontal bar (-) shows the median value. Vertical lines above or below the box indicate the corresponding quartile value (Q3 or Q1) plus or minus 1.5 times the interquartile interval (IQ = Q3−Q1).

The comparison of median and maximal values, presented in **Figure 6**, showed that both IgA and IgG were more efficiently recovered by the three-step method. Although the use of Jacalin gave better results, at least in the recovery of a single subtype of IgA, the superiority of the three-step procedure resided in its higher and more reproducible recovery of IgG antibodies. Fractioned elution by ionic exchange allowed the effective and quantitative separation of IgA sub-fractions, such as dimeric and monomeric IgA and J chains, as shown by the chromatogram presented in **Figure 5** **, panel C**. A difference in the relative concentrations of monomeric and dimeric sIgA was observed in the course of HPLC purification; however, the variability in concentration of the various IgA subtypes was not addressed in these experiments.

Presented results also confirmed that IgG are more abundant than IgA in the female and male genital fluids; in detail, the IgA1 subtype was found in both genital fluids. In detail, the mean IgG levels found in IgA-depleted fractions were 142.2 (range 45–321) vs mean IgA 72 (range 41.5–100) microg/sample (**Table 4**); their concentrations were in agreement with data previously reported. Total IgA were also evaluated in IgA1-depleted fractions, and the mean of total IgA was 15.5 (range <0.125–469) microg/sample. Similar results were obtained with seminal fluids (data not shown).

**Table 4:** Mucosal IgA and IgG, from female genital fluid.

	Sepharose-anti-Ig columns		Agarose-Jacalin column (IgA1)	
	IgA	IgG	IgA-enriched, i.e. anti-IgA	IgA-depleted, i.e. anti-IgG
**10 IT HC**	<0.125 (<0.125)	626.8 (135–1,444)	66.8 (41.5–100)	142.2 (45–321)
**10 IT HIV+**	43 (<0.125–125)	839.2 (130–1627)	93.3 (16.6–252.6)	807.6 (123–3,610)
**10 CA HC**			54.12 (29–85)	66.84 (15.6–123.9)
**10 CA HIV+**			49.62 (5.7–116)	376.52 (58.8–702)

IT: Italian; CA: Cambodian; HIV+: HIV seropositive; HC, healthy women. Immunoglobulins were obtained by immuno-affinity purification or by agarose-jacalin isolation.

Results are expressed as total micrograms, mean and range.

Either preserving procedures, chemical addition and other phases of immunoglobulins purification did not affect the avidity and specificity of antibody binding; indeed, immunoglobulins from HIV-positive specimens conserved their anti-HIV reactivity when assayed in commercial ELISA kits (data not shown). Method reproducibility on individual specimens was assessed on three individual samples (three female and three male genital fluids). Each specimen was split in three aliquots and the three series were processed with the optimized protocols in three different purification rounds (data not shown). Differences in IgG and IgA values were found not statistically significant.

### Method reproducibility: Italian vs Cambodian HIV cohorts

The vaginal fluids from a cohort including healthy females (not sex workers) and HIV-positive Cambodian female sex workers (n = 20) were purified and analyzed with the methods previously set up; results were compared to those obtained from the Italian cohorts of healthy donors and HIV-positive subjects (males and females) which were used to set up optimal assays conditions. This experiment was aimed at confirming the feasibility and reproducibility of our methods on a cohort of healthy and HIV-positive individuals endowed with a different genetic background.

Due to the local sampling practice, mucosal fluids from Cambodian women were obtained by cervico-vaginal lavage (CVL), and sandwich ELISA was performed as described in Protocol #6; however, mucosal antibodies were purified by the three-step chromatographic method previously described.

The mean IgA values were respectively 86.6 microg/mL (range: 8.4–262.16 microg/mL) for healthy women and 156.2 microg/mL (range: 30–379.2 microg/mL) for HIV-positive patients. Immunoglobulin values were lower than those observed in the Italian cohort, which were, 297.1 microg/mL (range: 16.6–687) and 682.9 microg/mL (range: 11.1–2,692.1 microg/mL), respectively. The methods applied, especially the protocol #6 for sandwich ELISA, confirmed their sensitivity also in this cohort, that showed a different immunogenetic background and a different responsiveness to infection. In fact, the comparison between Italian and Cambodian cohorts, summarized in **Table 4**, shows a lower antibody presence in vaginal fluids from the latter population.

## Discussion

Mucosal districts of the genital tract play a key role in protection from STD and HIV infection, due to their involvement in both horizontal and vertical disease transmission. The differences in the innate and adaptive mucosal responses could prove valuable in studying resistance to sexually transmitted infections and to HIV; however, many attempts achieved puzzling results and many findings failed to be further confirmed by scientific community. Indeed, mucosal fluid sampling and analysis often gave conflicting results, due to heterogeneity of cohort population enrolled in studies as well as to the lack of uniform methods for sampling and testing.

Large amounts of anti-HIV Igs, especially IgG, were usually found in sera from HIV-positive people, while mucosal immunoglobulins, especially IgA, were detected in some cohorts but not in other; however, even when observed, IgA were seldom reported to neutralize the virus [28]. Conversely, mucosal, neutralizing IgA were found in genital secretions from HIV-exposed, seronegative people (ESN); some studies failed in detecting such IgA. It is believed that the higher virus exposure might be related to a stronger mucosal response: in some cohorts, up to 70% of enrolled sex workers displayed neutralizing IgA. It is known that IgA concentration largely depends on genetic or environmental factors, as well as on the mode and frequency of exposure, fluid sampling or analytical techniques. Anti-HIV antibodies account for less than 5% of the total immunoglobulins, and only a portion of binding antibodies can actually block the virus; according to literature, neutralizing titers of HIV-specific immunoglobulins range from 1 to 600 microg/mL [17].

In genital fluids, antibodies have different concentrations than in serum; moreover, mucosal IgA are less abundant than IgG, that are provided from systemic response. Due to their paucity, IgA are therefore more prone than IgG to the risk of low recovery rates or to the reagent cross-reactivity during assays. Among IgAs, secretory IgA (S-IgA) is the dominant isotype in most mucosal secretions, and displays several advantageous features when compared to IgG and IgM [29]. Locally-produced S-IgA is composed of polymeric IgA associated with J chain and SC components, acquired during selective and active epithelial transcytosis [30]. Unlike IgG or monomeric IgA, S-IgA is especially resistant to endogenous and exogenous (bacterial) proteolytic enzymes, which are abundant in the GI tract, in the oral cavity, in the respiratory tract, as well as in CVL fluids and semen. Genital proteases promptly digest IgM and IgA monomers, but have little effect on S-IgA [31]. Due to their peculiar feature, IgAs have not only the potential to neutralize free viruses in sera and external secretions, but also to block viruses present within epithelial cells [30]. The specific role of mucosal IgA in HIV protection is still under discussion, and the marked differences observed among various cohorts and in different laboratories undoubtedly contributed to confusion. The lack of standardized methods to investigate mucosal compartments is a main reason, if not the major one, that weakens the resolution of the debate. In fact, the definition of protective responses to HIV requires specific know-how and experience as well as tools and methods able to achieve solid and reproducible results in more than a single laboratory.

In vitro, IgA reactivity may be affected by components of mucosal fluids, e.g. mucin and glycoproteins, resulting in altered quantitation and purification procedures; due to their lower concentrations, mucosal IgA are more sensitive to contaminants than IgG.

This study has systematically examined all phases of immunoglobulins purification, from sampling to detection and quantitation, has compared different methods and has considered each step in detail, with the aim of setting a specific, standardized and reproducible method to investigate mucosal antibodies and especially IgA. The key point of the method here described can be briefly summarized in the use of Weck-cel sampling for vaginal secretion; the immediate processing of fresh semen to separe cell fraction, and the addition to both genital fluids of EDTA and antibiotics to prevent bacterial and/or enzymatic degradation; protease inhibitors were found more effective in semen processing and did not increase the recovery significantly. Three-step HPLC column purification achieves quantitative and reproducible IgG and IgA purification, as well as the fractioning of mono- and dimeric IgA; further affinity purification with jacalin can split the IgA1 and the IgA2 subfractions. **Table 5** summarizes in detail the optimal methods for all phases of purification.

**Table 5:** Advices for working with human mucosal specimens and for recovering the highest level of mucosal immunoglobulins.

	*Seminal fluids*	*Vaginal fluids*	*Both genital fluids*
Sampling	---	Weck-Cel strips	---
Processing	IgG: Immediate addition of IP+AB	IgG: Immediate addition of IP+AB	---
	IgA: Immediate addition of EDTA+IP+AB	IgA: immediate addition of EDTA+AB	---
Detection by Sandwich ELISA	---	---	Overcoating: Skimmed Milk 1%
	---	---	Detection with biotinylated anti-human Igs and Streptavidin-HRP
Three-step chromatographic purification	---	---	IgG: Sepharose-Protein-G column
	---	---	IgM+IgA: Anion exchange
	---	---	IgA subtypes: Gel filtration
	---	---	IgA1 subtypes: Agarose-Jacalin column

Sampling method is determinant to obtain a sufficient quantity of mucosal fluids. Weck-Cel method was superior to cervico-vaginal lavage (CVL) and to brushing in recovering the highest amount of fluid and therefore of antibodies [20], [21].

The “immediate” fluid processing was more effective than the “delayed” mode in preserving antibodies, and was superior to simple cryoconservation for both genital fluids. In vaginal specimens, IgG antibodies resulted better protected by the “immediate” addition of single or double additives containing antibiotics and/or EDTA; the triple additive was also effective, and maintained its efficacy after model adjustment. Interestingly, the addition of antibiotics and EDTA was associated to an higher IgA concentration. The different influence of additives could reflect a different sensitivity to proteolysis of IgG and IgA, and suggested a major role for bacterial flora in respect to proteolytic enzymes from vaginal fluids. In seminal samples, the triple addition of antibiotics, EDTA and protease inhibitors significantly improved IgA recovery in respect to the single and double additives. The superior efficacy of the triple additive was also confirmed in the “delayed” processing mode, whereas the optimal method for seminal IgG consisted in the double addition of antibiotics and protease inhibitors. The standard method recommended in current protocols, tested in “delayed mode” as a positive control, was uniquely found superior to the negative control method, i.e. cryoconservation [23]. HIV serostatus did not affect antibody recovery by a chemico-physical point of view; rather, the fluids from HIV-positive individuals contained lower quantities of IgA antibodies than those from healthy donors, probably due to changes in immunity caused by virus infection and replication.

Sandwich ELISA assay offers higher sensitivity in detecting mucosal antibodies, that are in lower amount than serum immunoglobulins [19]. Assay sensitivity is a key issue for mucosal IgA, that are in lower concentration than mucosal IgG. Due to these reasons, the study set up a two-step method for amplification and detection (Biotinylated-Igs and Streptavidin-HRP), that provided higher sensitivity in respect to the direct detection with peroxidase-conjugated jacalin. The milk-based overcoating buffer controlled aspecific binding more effectively than the use of BSA and detergents. Conversely, conjugated anti-sera to human Igs not only detected mucosal antibodies under assay, but also weakly cross-reacted with other antibody types (Table 3).

Affinity purification by jacalin-agarose offered quantitative and reproducible recovery of the unique IgA1 fraction from genital specimens; either the one- or the two-step immunoaffinity columns, previously shown to work well with serum antibodies, were poorly manageable in mucosal IgA purification [32]. The three-step affinity purification was superior to the other protocols assayed, despite its higher number of steps. Three steps could appear time-consuming and might suggest a potential waste of molecules or a higher risk of contamination, but it was not the case. Indeed, the advantage of the method resides in the resolution of IgA subfractions, as the monomeric, dimeric IgA and the secretory component were easily separated and recovered. A limit of the three-step protocol, the lack of discrimination between IgA1 and IgA2 subtypes, can be overcome by a further separation step, carried out on a Jacalin-agarose column.

All methods and protocols set on the Italian cohorts were applied to the analysis of a Cambodian cohort, including healthy women and HIV-positive patients, with the aim of evaluating the whole procedure on a different study population, endowed with a different genetic background. Antibody concentrations were highly different among individuals as well as between the Italian and Cambodian populations. Both the mean and range values for IgG were higher in HIV-positive patients, while the mean IgA concentrations were similar, but highly variable, being distributed over a wider range of individual values than in the Italian cohorts. Both IgA and IgG concentrations were lower in Cambodian fluids than in the Italian ones, irrespective of the HIV serostatus. The sandwich ELISA assay described in the study resulted feasible to be applied in developing countries settings, due to its high sensitivity, to the ease of standardization and to the low cost.

### Conclusion

Taken together, methods set up in this study allowed to obtain immunoglobulins of both IgG and IgA types from specimens of male and female genital fluids, showing to be feasible and reliable tools for investigation of genital mucosa in general and/or of specific local immunity to HIV. Methods here described were successfully used in different study cohorts, confirming their solidity, because individual or ethnic differences in immune responsivity or humoral reactivity to infections did not affect significantly the analysis. Without specific, standard methods for measuring mucosal immunoglobulins, immune investigation lacks of basic tools to study and compare local immunity, and HIV research is severely hampered in one of its major aims, i.e. identify and reproduce protective responses to block HIV infection just at virus's major portals of entry.
